# Insecticide resistance evolution and assisted gene flow interact to shape the evolution of plasticity

**DOI:** 10.1098/rspb.2026.2085

**Published:** 2026-11-11

**Authors:** Eric G. Prileson, Bianca Campagnari, Brynne Ruotsalainen, René S. Shahmohamadloo, Cristian Zetina, Seth M. Rudman

**Affiliations:** ^1^ School of Biological Sciences, Washington State University - Vancouver Campus, Vancouver, WA, USA; ^2^ University of California Riverside, Riverside, CA, USA

**Keywords:** phenotypic plasticity, evolution of plasticity, evolutionary rescue, rapid adaptation, insecticide resistance, assisted gene flow

## Abstract

Adaptive phenotypic plasticity can bolster fitness in changing environments, but the extent to which plasticity evolves rapidly, and which forces shape its evolutionary trajectory, are largely unknown. To empirically study the evolution of plasticity, we first conducted a replicated field experiment in which *Drosophila melanogaster* populations adapted to insecticide exposure and a subset of these populations received high diversity assisted gene flow. We then reared individuals from each population across temperature and insecticide treatments in common garden conditions to test the following questions: (i) has prior selection and rapid adaptation of insecticide resistance led to evolved shifts in plasticity relative to naive populations? (ii) Does gene flow from genetically diverse populations contribute to adaptive plasticity evolution relative to gene flow-restricted low diversity populations? Both gene flow and prior evolution of resistance influenced the evolution of plasticity for multiple traits and were often maladaptive for resistant and gene flow-restricted populations, suggesting a trade-off between trait and plasticity evolution. Assisted gene flow minimized maladaptive plasticity potentially through relaxation of underlying epistatic or pleiotropic constraints. Together, these results demonstrate the dynamic interactions between trait evolution, the evolution of plasticity and forces that shape genetic diversity, with implications for conservation of threatened populations.

## 1. Introduction

When environmental conditions shift over short time-scales, they can exacerbate mismatches between organismal phenotypes and the environment. In the absence of compensatory mechanisms that reduce this mismatch, populations are likely to decline, potentially to extinction. To persist, populations can respond across generations via evolution but also more immediately within a generation through phenotypic plasticity—the capacity of a genotype to express different phenotypes in response to environmental variation (figure 1*A*) [1,2]. Following environmental shifts, these environmentally dependent traits could be adaptive if the plastic response moves the phenotype closer to new fitness optima [3–5]. On the other hand, plasticity might be maladaptive if the direction of plastic response opposes novel fitness optima or fails to minimize the phenotype–environment mismatch [6,7]. Complicating this picture is the fact that across generations, plasticity itself can evolve rapidly, with potentially strong effects on fitness (figure 1*A*) [1,8,9]. Determining the pace, direction and key drivers of the evolution of plasticity relative to trait evolution is therefore critical to understanding its role in population responses to environmental change, including potential rescue from extinction.

**Figure 1.**
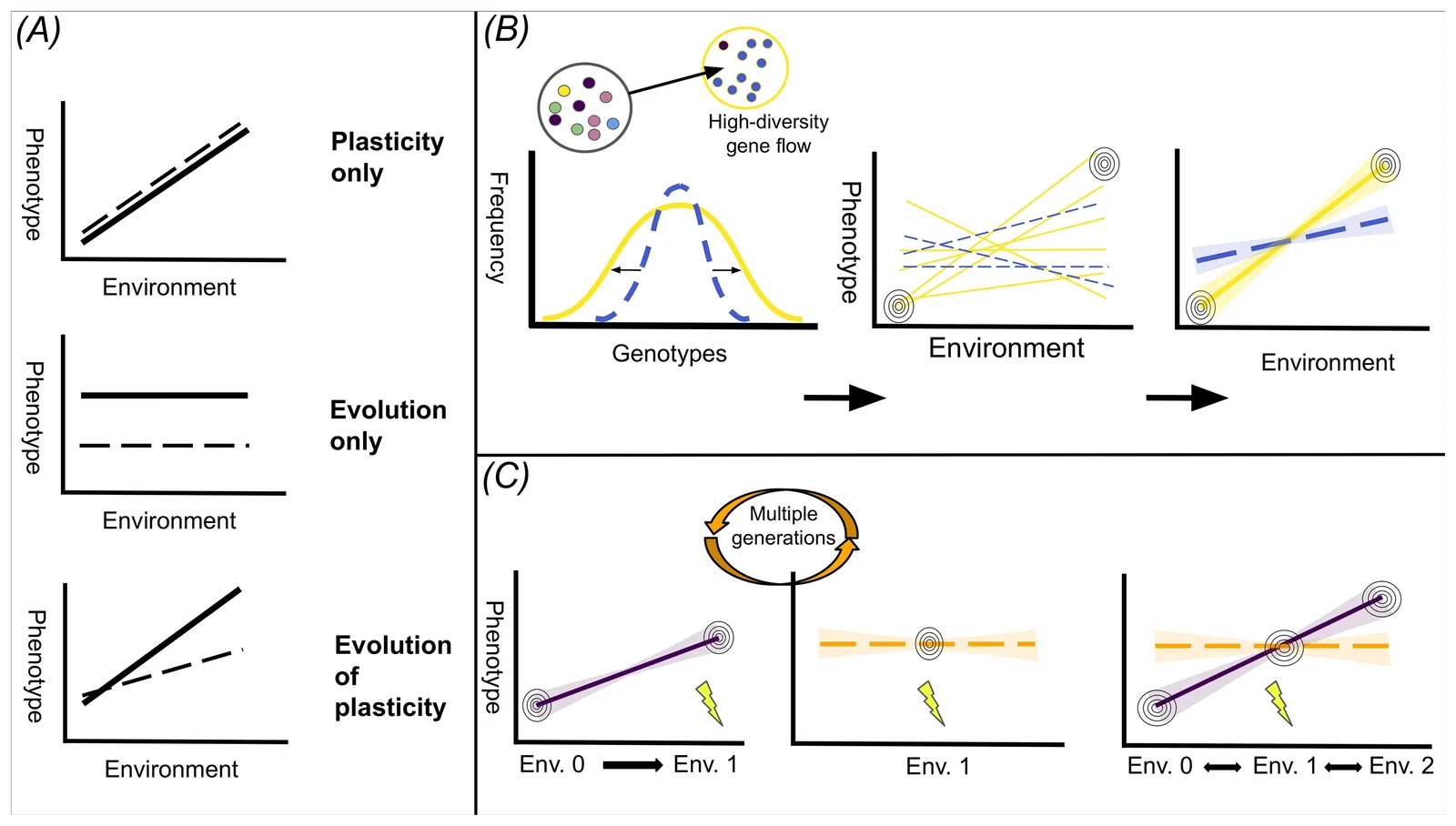
A simplified schematic of how linear reaction norms might differ between populations and processes underlying their evolution. *(A)* Three possible outcomes for population-level reaction norms across environments. Different populations (solid and dashed lines) can both express strong plastic traits (top), evolved differences with different intercepts but with no change in slopes (middle), or different intercepts and slopes (bottom). *(B)* The effect of genetic variation on the evolution of plasticity. An increase in the genetic diversity of a population, such as through assisted gene flow (left), can provide greater variation in reaction norms in diverse (solid lines) relative to less diverse (dashed lines) populations. For genetically diverse populations (centre; solid lines), there is a higher probability of reaction norms within the population matching optima across environments (concentric circles) compared with less diverse populations (dashed lines) and selection can move the average reaction norm closer to these optima (right). *(C)* Depiction of genetic assimilation. A population can express an adaptive plastic response between an ancestral environment (Left; Env. 0) and a novel one with a strong stressor (Env. 1; lightning bolt). If the novel stressor remains and the maintenance of plasticity is costly, strong selection (centre) could canalize the expression of the phenotype such that the plastic response is lost over generations (orange dashed line). If the canalized trait does not match optima under returns to ancestral (right; Env. 0) or new environments (Env. 2), this could lead to a trade-off compared with a plastic population (purple solid line).

Plasticity can evolve if there is standing genetic variation in sensitivities to the environment (i.e. reaction norms), a process called genetic accommodation (figure 1*B*; [1,10,11]). Fundamental evolutionary forces that alter genetic diversity then are likely to be key for the evolution of plasticity [12,13]. For example, within sub-populations, selection can decrease, whereas gene flow can increase genetic diversity on which selection can act relative to the broader metapopulation [14,15]. Assisted gene flow, or the directed introduction of new alleles, is one such process that can increase the diversity and probability of persistence for populations at risk of extinction [16,17]. There is conflicting evidence, however, regarding whether increased genetic variation on its own will increase the magnitude of plasticity [18–21] or if evolved plasticity will be adaptive because gene flow could swamp locally adapted populations with maladaptive alleles [22]. In addition to potential effects of demography, the evolution of plasticity could also be constrained by costs of plasticity and maladaptive reaction norms [20,23,24]. Further, plasticity could also be lost altogether if selection canalizes previously plastic traits through genetic assimilation (figure 1*C*; [25,26]; reviewed in [27]). Thus, while genetic variation can lead to the evolution of plasticity, complexities of the fitness landscape will dictate the change in direction or magnitude of population-level reaction norms and their potential to rescue populations.

Natural populations face complex environments including anthropogenic stressors, but it is unclear how adaptation for one stressor bolsters or constrains the evolution of plasticity in multiple environments [28,29]. Spatial or temporal decoupling of a stressor in different environments presents an opportunity to test for evolutionary constraints or plastic phenotypes that are otherwise cryptic [30,31]. For example, temperate insects in an agricultural environment contend with both insecticides and seasonal variation, but because insecticides are primarily applied during warm seasons, low temperatures and insecticide exposure might not co-occur. Energetic constraints then may lead to selection favouring alternative alleles at different time points owing to negative trait correlations (via pleiotropy or linkage) that oppose the direction of selection [32,33]. Indeed, prior work documenting greater extinction of resistant *Drosophila melanogaster* populations over winter [34] could be explained by a trade-off between resistance evolution and adaptive thermal plasticity. In addition, simultaneous exposure to both insecticide and low temperature could dramatically affect reaction norms and reveal interactions that are antagonistic or synergistic, with the combined effects cancelled out or amplified, respectively [35,36]. Given these different outcomes, there is a clear need to investigate how resistance evolution impacts the evolution of plasticity in multiple environments, but to do so requires substantial population-level replication and a system that allows for experimental manipulation.

The fruit fly *D. melanogaster* (Meigen 1830) is an excellent system in which to test the evolution of plasticity and to identify the factors underlying it. As a model system, *D. melanogaster* is amenable to large-scale evolution experiments that provide environmental realism without reducing replication [37,38] and it has been used to track rapid adaptation over seasonal time-scales (i.e. adaptive tracking [39]). Further, the genetic resources of *D. melanogaster* allow for manipulation of genetic variation from a known genetic history. There is also abundant empirical evidence of developmental plastic responses in thermal physiology (reviewed in [10,40]), as well as genetic variation of plastic responses to low temperature [41–43]. When reared at low temperatures, *D. melanogaster* exhibits plastic traits that are putatively adaptive, including reduced fecundity [44,45], improved starvation tolerance [46], greater cold tolerance [47,48] and larger body sizes [49,50]. In addition, *D. melanogaster* can evolve resistance to insecticides [51–53], but it is unknown how resistance evolution impacts the evolution of plasticity. Sudden environmental stress such as insecticide exposure can lead to genetic assimilation in *D. melanogaster* ([25]; reviewed in [54]), but whether resistance is related to initial plasticity to insecticide exposure that becomes canalized is uncertain.

Here, we address how prior selection and assisted gene flow affect the evolution of plasticity in *D. melanogaster* by asking the following questions: (i) Does rapid evolution of insecticide resistance impact the evolution of plasticity relative to susceptible control populations? (ii) Within insecticide-resistant populations, does gene flow from a high-diversity population facilitate the evolution of adaptive plasticity relative to populations without gene flow? To test these questions, we used populations of *D. melanogaster* that differed in prior-evolved insecticide resistance and gene flow histories. Following evolution during an outdoor growing season and common garden rearing, we raised populations across temperature, insecticide exposure, and their combination to test the evolution of plasticity in stress response and fitness-associated phenotypes. Broadly, we expected directional selection and gene flow to shift the magnitude and direction of reaction norm slopes based on changes to genetic diversity [10,26], but we had divergent predictions for reaction norm evolution depending on the population and developmental environment. We predicted that across temperature, constraints from selection (for resistant populations) and reduced genetic diversity (from lack of gene flow) would lead to reaction norms that were putatively maladaptive. To test whether prior selection from insecticide leads to canalization, we predicted that across an insecticide treatment, resistant populations would have dampened plasticity but greater evolved trait values on the insecticide than susceptible populations. Together, these questions and our approach addressed how key processes affect the evolution of plasticity in complex environments with broader implications for responses to rapid environmental change.

## 2. Methods

### (a) Multigenerational experiment and founding fly populations

To investigate the evolution of plasticity, we measured plasticity in fly populations following a multigenerational outdoor selection experiment in 2024 in Vancouver, WA, USA. *D. melanogaster* populations were founded from DGRP lines (*Drosophila* Genetic Reference Panel; [55]). Mesocosms were maintained at the Washington State University Vancouver campus orchard (45.729 N, −122.633 W; henceforth ‘orchard’) from 18 June to 24 October 2024, encompassing nine overlapping generations. Throughout the experiment, all populations were fed a modified Bloomington recipe following Rudman *et al.* [39].

We experimentally manipulated the genetic composition of the starting *D. melanogaster* populations. We first founded a high-diversity population from 100 DGRP laboratory fly lines (henceforth ‘HD control’) and low-diversity populations from 15 DGRP lines (henceforth ‘LD control’). Each of these population types was recombined in the laboratory and reared for four generations on control medium prior to outdoor rearing. Additionally, a subset of the LD control populations was reared on medium treated with a 0.0375 µg l^−1^ concentration of the organic insecticide spinosad (Entrust; Dow AgroSciences, Indianapolis, IN, USA) as part of an artificial selection experiment for five generations (henceforth ‘pre-adapted’ flies). This concentration corresponds to the lethal dose at which 50% of flies experienced mortality based on dose–response assays conducted on founder flies under standardized laboratory conditions (see supplementary material in [53]).

### (b) Selection event and gene flow manipulation

We allowed populations to expand for one generation outdoors in 8 m^3^ mesocosms beginning on 18 June 2024. Then, on 5 July 2024, we introduced insecticide treatments to food media of 16 of the 36 LD control populations as a strong agent of directional selection (supplementary material, figure S1). For both the selection event and subsequent food treatments, insecticide medium was treated with a 0.0375 µg l^−1^ concentration of spinosad. Following two generations on the insecticide treatment, during which we observed strong mortality, we initiated gene flow treatments to test the influence of gene flow on rescue (see supplementary material, figure S1 and table S1, for details on timeline). In one of the insecticide-treated groups, we replaced 33% of the reproductive output with a matched number of eggs from the HD control population. This high diversity gene flow ‘HDGF’ treatment was conducted with six additions across 11 days to account for one *D. melanogaster* generation. This treatment simulated genetic rescue such that the influx of novel alleles from HD control populations increased total genetic diversity in the receiving cages. In a second insecticide-treated group, we again replaced one-third of the reproductive output but introduced ‘pre-adapted’ gene flow from the laboratory-adapted resistant population, such that gene flow probably increased the frequency of resistance alleles but not genetic diversity. We termed the population receiving pre-adapted flies as ‘PAGF’. For both HDGF and PAGF populations, we removed an equal proportion of eggs laid by the original population to keep the total population size consistent before and after gene flow treatment. This ensured that any change was attributable to gene flow (HDGF) or changes in frequency of pre-adapted alleles (PAGF) and not direct demographic or competitive effects.

A key contrast for the HDGF population was a treatment founded with the same low-diversity populations that did not receive gene flow (we termed these ‘NGF’ for no gene flow). NGF flies that survived exhibited resistance to spinosad only via adaptation from standing genetic variation—an example of evolutionary rescue [56,57]. Collectively, based on survival when reared on insecticide media, we refer to the NGF, PAGF and HDGF populations as ‘resistant’ to spinosad. Because we did not manipulate genetic diversity through gene flow with PAGF populations, we pooled NGF and PAGF populations together as a ‘resistant’ group that we could compare with unexposed ‘LD control’ populations that had the same pool of genetic diversity. For the remainder of the growing season (approx. seven generations), resistant flies continued to be exposed to spinosad medium at the same dosage as the initial exposure.

### (c) Collection and initial common garden rearing

After nine overlapping generations near the end of the growing season (6 and 24 October 2024; supplementary material, figure S1 and table S1), we collected approximately 800 eggs from each field population in density-controlled 200 ml plastic bottles from 10 LD control, 10 HD control, 8 NGF, 8 PAGF and 8 HDGF cages. We then reared flies indoors under common garden conditions at 25°C under 12 : 12 L : D lighting and 40% humidity for three generations to control for density and environmental effects among outdoor populations. We were able to successfully rear flies from eight LD control, four NGF, seven PAGF and four HDGF populations. Four of the original eight NGF and HDGF populations had insufficient population size for accurate sampling and were excluded. Upon eclosion of the third generation reared under common conditions, we collected 1500 eggs/300 ml, a standard non-stressful density for the developmental plasticity experiment.

### (d) Laboratory rearing and full factorial design

We transferred freshly laid eggs to media vials at a standard low density (30 eggs/vial) in one of four conditions: (low temperature (18°C), control medium; low temperature, spinosad medium; warm temperature (25°C), control medium; warm temperature, spinosad medium); supplementary material, figure S1). At warm temperatures, flies developed in approximately two weeks and were monitored for eclosion and per cent survival. Flies developing at low temperature took approximately two weeks longer and thus phenotyping occurred in two non-overlapping time periods (supplementary material, table S1). Although 18°C is a non-stressful temperature for *D. melanogaster*, it can induce differential gene expression and phenotypes [58,59]. Three to five days following eclosion, we conducted our phenotypic assays for all combinations.

### (e) Phenotypic assays

The following phenotypes were assayed per replicate population: (i) insecticide resistance measured as survivorship to adulthood: the proportion of eggs (30 eggs per vial) that survived to adulthood in three replicate vials; (ii) fecundity: the total eggs laid by a group of five females over 3 days, measured daily in each of three replicate bottles; (iii) starvation tolerance: the time to starvation for three replicate vials containing 10 males each on agar-only medium; (iv) adult body size, measured as the average dry mass of three pools of five females, dried at 55°C for 24 hours; (v) we also included chill coma recovery time (CCRT), a static measure of cold tolerance widely used in assessing low temperature responses in *D. melanogaster* [47]. As flies reared at 18°C largely did not experience chill coma at 4°C (supplementary material, table S2), we excluded this group from the cold tolerance assay and so CCRT was only compared among populations reared at 25°C across media treatments.

Determining when phenotypic reaction norms are adaptive or not is challenging because phenotypic responses may or may not affect fitness [60,61]. As we did not measure direct effects to fitness, we use the language ‘consistent with adaptation’ or ‘putatively (mal)adaptive’ and suggest that inferences of whether or not reaction norm evolution was truly adaptive be interpreted with caution. Nevertheless, trait performance in ectotherms including *D. melanogaster* can be strongly temperature dependent [62] and the degree of deviation from trait levels and expectations from thermal performance curves can be a proxy for fitness [4,63]. We therefore considered the plasticity of one population to be adaptive relative to another when reaction norms matched the magnitude and direction predicted by *D. melanogaster* and insect thermal physiology (supplementary material, table S3), although we note that these proxy measures serve as guidelines rather than hard and fast rules.

### (f) Statistical analyses

Plasticity can be modelled as the linear change in means across environmental treatments (i.e. the reaction norm slope [64–66]) and we follow similar empirical approaches that approximate the reaction norm at the population level [67,68]. To model each phenotypic measure for each question, we used generalized linear mixed-effects models constructed with the *glmmTMB* function from the *glmmTMB* package [69]. Since the populations were common garden reared for three generations prior to developmental treatments, we consider the between-population variation in plastic responses to be owing to genetic differences, not within-generation environmental exposures, and that they represent evolved differences in plasticity ([65,70]; but see [71]). For each phenotypic response, a significant effect of population type, treatment condition (temperature and/or medium treatment), or their interaction would indicate evolution between populations, plasticity, or the evolution of plasticity, respectively.

For our first question of resistance evolution and plasticity, we sought to test the interaction between population type, medium treatment and temperature on phenotypic response. At low temperatures and insecticide treatment, however, mortality was much higher for control populations (supplementary material, table S4). This reduced sample size prevented the comparison across both medium treatment and temperature for all control population phenotypes except resistance. We therefore constructed models with phenotype as the response variable, and either temperature, population and their interaction as the predictor variables, or separately, with medium treatment, population and their interaction as predictor variables. To test our second question of gene flow history and plasticity, we included medium treatment, population type and temperature in our models (with the exception of CCRT). Therefore, within each model, each phenotype was the response variable and temperature, medium treatment and population type were the fixed effect predictor variables, along with their pairwise interactions. For both questions, all models included individual population (cage) as a random intercept (see supplementary material for details on each model).

The total variance in reaction norms rather than the population level mean could be a key indicator of genetic variation of plasticity [66,72] and model results indicated potential differences in reaction norm variances between populations. We therefore ran Levene's test for heteroscedasticity using the *leveneTest()* function from the *car* package, but found no differences in variance (supplementary material, table S5).

We conducted model diagnostics using the *simulateResiduals* and *testDispersion* functions from the *DHARMa* package [73] and determined significance of the predictors using log-likelihood ratio tests in the *Anova* function with the type = ‘III’ argument from the *car* package [74]. When interaction terms were significant, *post-hoc* tests were conducted to identify differences between treatment levels and populations and were corrected for multiple comparisons using the *adjust = ‘fdr’* argument in the *emmeans* function from the *emmeans* package [75]. All analyses were conducted in R v. 4.4.0 [76].

## 3. Results

As insecticide but not temperature served as both the agent of field selection and a treatment during development, we clarify the results into three distinct patterns of the evolution of plasticity: (i) thermal plasticity—a population-level difference in reaction norm across temperature; (ii) insecticide plasticity—a population-level difference in reaction norm across medium treatment. For most phenotypes, this reflects a novel developmental response but not the resistance phenotype that instead reflects the same selective history that defines the resistant populations; and (iii) environment-dependent plasticity—population-level differences in reaction norms during development in both treatments where reaction norm differences were dependent on both environments (i.e. a three-way interaction).

### (a) Insecticide resistance and plasticity

The vast majority of flies developed successfully in three of the four treatments, but when exposed to the combination of low temperature and the insecticide, only 1.4% of flies in control populations survived to adulthood (supplementary material, table S4). With this reduced sample, we could only test the evolution of plasticity in the combined low temperature and insecticide treatment for the resistance phenotype (i.e. survival when exposed). For the remaining phenotypes, we considered evolutionary divergence of plasticity across temperature and media treatments separately.

We found that plasticity of resistance evolved between resistant and control populations, with a significant three-way interaction between population type, temperature and insecticide treatment (figure 2*A*; estimate = 14.4 ± 5.39 s.e., $\chi^{2}$ = 7.15, *p* = 0.00751). This result indicates that both thermal plasticity and insecticide plasticity diverged between populations, but also that they were environment-dependent. This pattern was driven by considerable mortality for all populations when reared at low temperatures, but only when also developing on insecticide medium (supplementary material, table S6). Although survival on spinosad declined at the cooler temperature, resistant populations still maintained higher survival than control populations (supplementary material, table S7).

**Figure 2.**
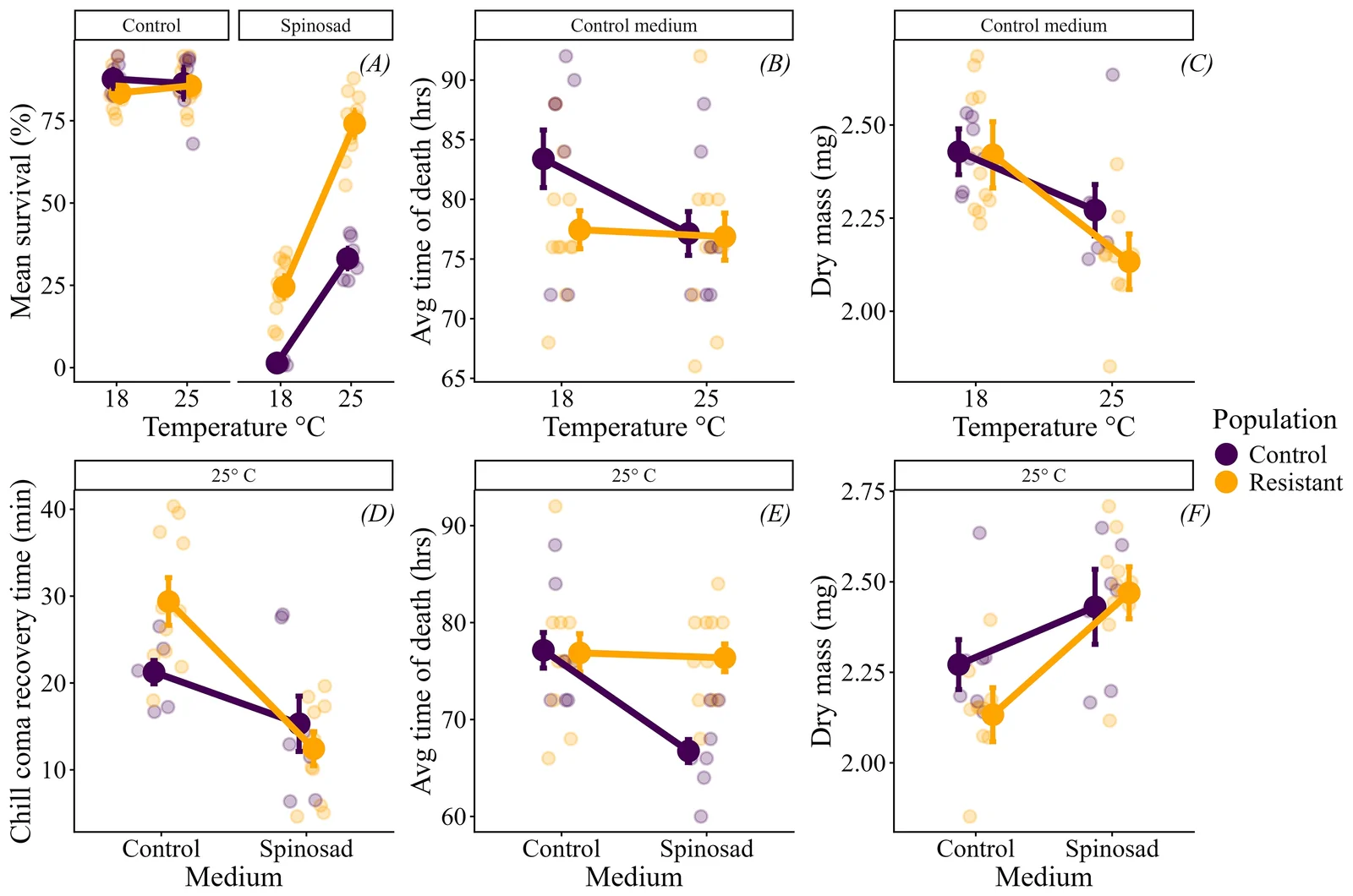
Phenotypic responses of control and resistant populations of *D. melanogaster* to temperature (top row) and media treatments (bottom row). Large points show the mean trait value of each population type ± 1 s.e. Smaller points jittered behind the means show raw data values for each independent population's phenotypic measure. *(A)* Spinosad resistance measured as the per cent egg-to-adult survival on insecticide media (note: inset panels denote media treatments). *(B)* Starvation tolerance measured as average time to mortality. *(C)* Average body size measured as dry mass in milligrams. *(D)* Cold tolerance measured as chill coma recovery time in minutes. *(E)* Starvation tolerance measured as average time to mortality across media treatment at 25°C. *(F)* Average body size measured as dry mass in milligrams.

#### (i) Responses across temperature

Across temperature on control medium, thermal plasticity evolved for starvation tolerance and body size with significant interactions between population type and temperature (figure 2*B*,*C*; supplementary material, tables S6 and S7). Control populations exhibited stronger plasticity, with improved starvation tolerance at low temperature (control—resistant at 18°C = 6.26 ± 3.06 s.e., *z* ratio = 2.04, *p* = 0.0409), whereas in resistant populations, low temperature did not induce a response (18 − 25°C = 0.559, ±2.93 s.e., *z* ratio = 0.191, *p* = 0.851). By contrast, body size declined at a greater rate in response to warmer temperatures for resistant populations, such that they exhibited a smaller size at 25°C relative to control (control − resistant 25°C: estimate = 0.142 ± 0.0540 s.e., *z* ratio = 2.63, *p* = 0.00860). We did not find evidence for the evolution of thermal plasticity for fecundity, but as expected, we did see a strong plastic effect of temperature on fecundity (supplementary material, figure S2; estimate = 18.2 ± 1.90 s.e., $\chi^{2}$ = 92.2, *p* < 0.0001).

#### (ii) Responses across insecticide

Across insecticide exposure at 25°C, there was evolutionary divergence in insecticide plasticity between populations for CCRT, starvation tolerance and body size, with a significant interaction between population type and insecticide treatment (figure 2*D*–*F*; supplementary material, tables S6 and S7). For CCRT, both populations showed faster recovery time on spinosad medium (estimate −6.95 ± 1.15 s.e., $\chi^{2}$ = 8.06, *p* = 0.00452), but resistant populations exhibited stronger negative plasticity with a longer recovery time on control medium (figure 2*D*; control − resistant CCRT, control medium: −7.06 ± 3.44 s.e., *z*.ratio = −2.05, *p* = 0.0403). Control populations exhibited strong declines in starvation tolerance when exposed to spinosad, whereas resistant populations showed little plasticity (figure 2*E*; control − insecticide, control population = 10.3, ±2.92 s.e., *z* ratio = 3.52, *p* = 0.000400; resistant = 0.182, ±2.33 s.e., *z* ratio = 0.0780, *p* = 0.938) and maintained greater starvation tolerance (supplementary material, table S6). Control populations also expressed a non-significant pattern of greater decline in fecundity when exposed to insecticide (supplementary material, figure S3 and table S7) but resistant populations exhibited a greater increase in body size from a smaller starting point on control medium (figure 2*F*; resistant – control = −0.142 ± 0.0501 s.e., *z* ratio = 2.83, *p* = 0.00460).

### (b) Effect of gene flow on plasticity

Gene flow from the high diversity population influenced the evolution of thermal and insecticide plasticity for fecundity, starvation tolerance and CCRT, with significant interactions of population type, temperature and/or medium treatment (figure 3*A*–C; supplementary material, table S8). Both populations exhibited higher fecundity as temperature increased, but the induced response was weaker for NGF populations on insecticide medium (figure 3*A*; supplementary material, table S9; HDGF − NGF 25°C = 5.47 ± 2.08 s.e., *z* ratio = −2.64, *p* = 0.00840). Starvation tolerance thermal plasticity evolved on insecticide but not control medium (figure 3*B*; supplementary material, tables S8 and S9), with greater starvation tolerance at 18°C and stronger thermal plasticity for HDGF populations across temperature compared with NGF populations (HDGF – NGF = −10.0 ± 3.66 s.e., *z* ratio = −2.73, *p* = 0.00630). Recovery from chill coma was faster on insecticide medium for both populations (figure 3*C*; CCRT estimate = −17.3 ± 0.864 s.e.; $\chi^{2}$ = 398.7, *p* < 0.0001) but on control medium, HDGF populations had a faster recovery time (supplementary material, table S9; HDGF – NGF = −10.0 ± 2.44 s.e., *z* ratio = 4.11, *p* < 0.0001).

**Figure 3.**
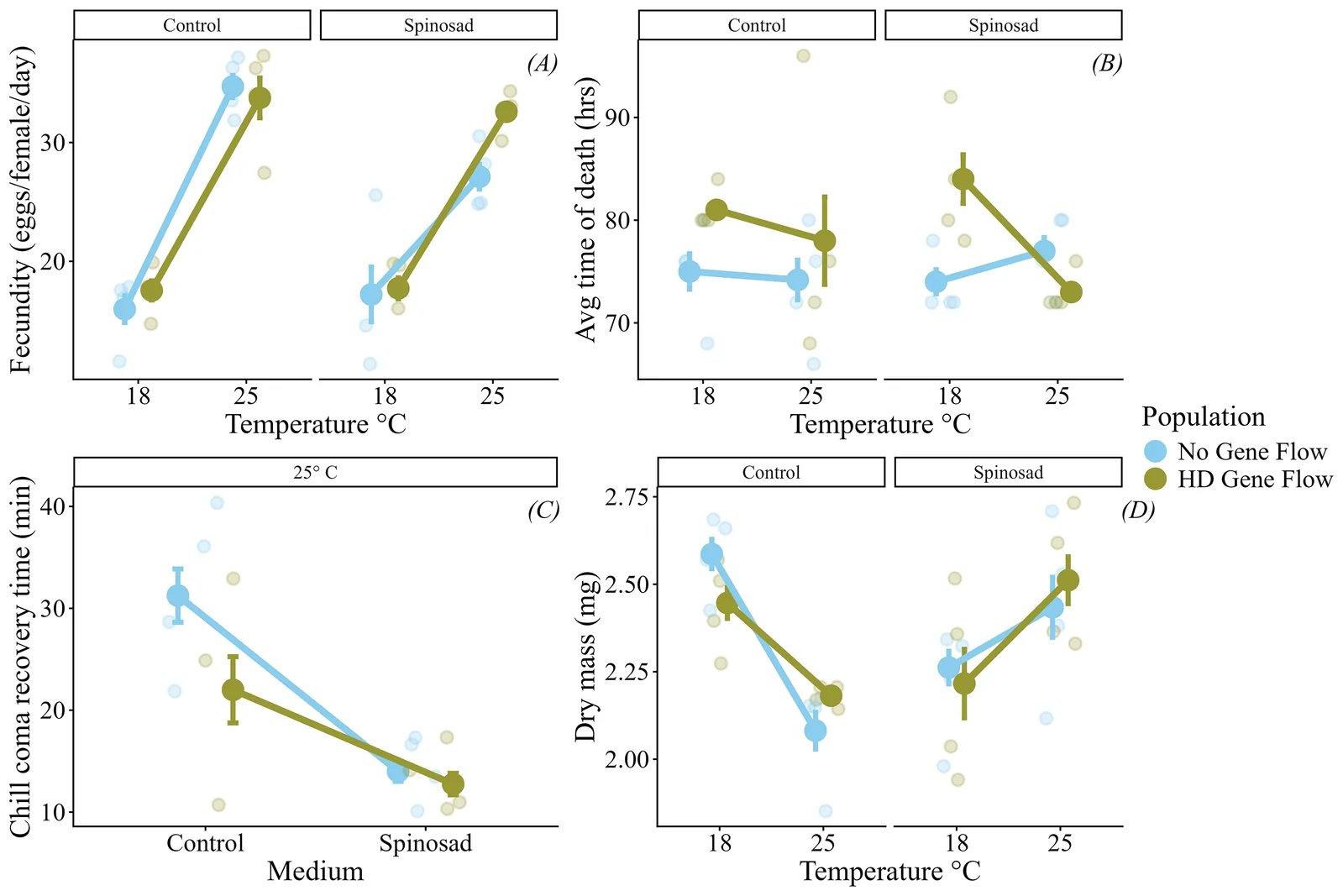
Phenotypic responses of *D. melanogaster* to temperature and media treatments (*x*-axis and inset panels) of resistant populations with divergent histories of gene flow: blue = NGF, gold = HDGF. Large points show the mean trait value of each population type ± 1 s.e. Inset panels denote media treatments. Smaller points jittered behind the means show raw data values for each independent population's phenotypic measure. *(A)* Average fecundity across a 3-day assay. *(B)* Starvation tolerance measured as average time to mortality. *(C)* Cold tolerance measured as chill coma recovery time in minutes. *(D)* Average body size measured as dry mass in milligrams.

For body size and resistance, there were plastic responses to temperature and insecticide, but reaction norms did not differ between population types within each treatment (figure 3*D*; supplementary material, figure S4 and table S9). As temperature increased, body size declined for both HDGF and NGF populations on control medium (18°C − 25°C NGF: 0.495 ± 0.0472, *z* ratio = 10.9, *p* < 0.0001; 18°C − 25°C HDGF: 0.257 ± 0.0485, *z* ratio = 5.52, *p* < 0.0001), but on insecticide medium, the direction of the plastic response was reversed (18°C − 25°C no gene flow: −0.200 ± 0.0519, *z* ratio = −4.001, *p* < 0.0001; 18°C − 25°C HDGF: −0.296 ± 0.0485, *z* ratio = −6.35, *p* < 0.0001). For resistance, both populations had high survival on control medium regardless of temperature, but on insecticide, resistance declined sharply at 18°C (supplementary material, figure S4 and table S9).

Last, we evaluated the extent to which gene flow from HD control populations reflected reaction norm variation in HDGF populations rather than post-gene flow divergence. We found that HD control populations exhibited similar reaction norms to HDGF populations (i.e. non-significant interaction terms) for most phenotypes (body size, fecundity, starvation tolerance) but they did differ in CCRT and resistance (supplementary material, tables S10 and S11). Although resistance was greater for HDGF populations as expected, surprisingly, HD control populations exhibited longer CCRT.

## 4. Discussion

Adaptive plasticity can be a critical component of how species respond and persist in the face of global change. Despite the potential importance of adaptive plasticity for the preservation of biodiversity, little is known about how plasticity evolves in the complex environments typical for many natural populations. We find that plasticity does evolve rapidly in fewer than 10 generations. Moreover, both gene flow and selection can affect the magnitude and direction of plasticity evolution, and for some phenotypes, the prior adaptation of insecticide resistance or lower diversity led to shifts that were putatively maladaptive. Together, these findings offer insight into how plasticity might evolve rapidly in fluctuating natural conditions, with implications for both agriculture and conservation.

In the absence of gene flow, evolutionary rescue can proceed from standing genetic variation, but demographic costs of selection might inhibit rescue or can leave populations less capable of longer-term persistence (reviewed in [77,78]). In our study, we tested how prior selection and adaptation for one stressor, an insecticide, affected the evolution of plasticity to a second unrelated environmental stressor, temperature. Although plasticity is expected to evolve more readily when stress responses are positively correlated, we found that both prior evolution of insecticide resistance and high-diversity gene flow influenced the evolution of thermal and insecticide plasticity, with shifts in reaction norm slope magnitude and/or direction. Although the evolved shifts were not always in the predicted magnitude or direction, this confirmed our expectation that broad-level evolutionary processes can drive the evolution of plasticity. Reaction norms for resistant and NGF populations in some traits were dampened and putatively maladaptive relative to control and HDGF populations, respectively. The influx of alleles provided by the high-diversity gene flow treatment, however, generally alleviated maladaptive plasticity, providing empirical evidence that assisted gene flow can contribute to the evolution of adaptive plasticity and play an integral part in evolutionary rescue. We delve into the specific evolutionary shifts in reaction norms for each question below and discuss the broader implications for these results.

### (a) Resistance evolution and the evolution of plasticity

We had predicted that prior selection from insecticides would influence the evolution of plasticity and this was supported as there were shifts in reaction norm slope magnitude and direction between control and resistant populations. Specifically, there were shifts in reaction norms for starvation tolerance and body size across temperature, as well as in fecundity and cold tolerance across insecticide treatment (figure 2*B*–*D*; supplementary material, figure S3). Since resistant populations expressed worse cold tolerance on control medium and exhibited weaker starvation tolerance at the low temperature, this matched our prediction of a trade-off between resistance evolution and thermal plasticity. In addition, across insecticide treatment, resistant populations expressed dampened plasticity for fecundity and starvation tolerance, consistent with our hypothesis of canalization. The evolved shifts in reaction norms, however, did not always match our predictions. Plastic responses for body size, for instance, were stronger in resistant relative to control populations and not consistent with maladaptation, suggesting that the adaptive nature of these shifts, and if plasticity is lost, is phenotype dependent.

Resistance to insecticides can evolve through many pathways and understanding this requires consideration of how a multifaceted genetic architecture might interact with the environment [79,80]. Consistent with this idea, spinosad resistance in *D. melanogaster* is probably polygenic [80,81] and reflects broad spectra of shifts in allele frequencies, including (either directly or indirectly) alleles related to thermal reaction norms. For example, allelic shifts underlying resistance potentially include pleiotropy or linkage that could explain the negative correlation between resistance and cold and starvation tolerance reaction norms [30,82]. Further, the intense exposure to spinosad during the growing season potentially selected for non-plastic traits in resistant populations, either through canalization or because costs of maintaining plasticity were high relative to non-labile traits [23,27]. Exposure to spinosad can lead to sub-lethal effects in naive *D. melanogaster* [53,83,84] and we found evidence of these effects with lower evolved fecundity and starvation tolerance for control populations. Surprisingly, both control and resistant populations exhibited a beneficial plastic cold tolerance response when exposed to spinosad at 25°C, suggesting that development with spinosad at warm temperatures might elicit a general stress response needed for future recovery from chill coma [31,85]. Considering that development at low temperature and spinosad was detrimental to survival, any cross-protective effect of insecticide exposure seems to be limited when encountering seasonal cooling.

### (b) Gene flow and the evolution of plasticity

As expected, gene flow played a strong part in shaping the magnitude and direction of plasticity evolution. This was reflected in the largely parallel reaction norms between HD control and HDGF populations. This suggests that alleles from the donor population that increased genetic diversity provided a substantial basis for divergence, but that some of the variance probably occurred as a result of prior selection from spinosad exposure. In addition to divergence based on gene flow, for several traits, evolved shifts were putatively adaptive. Specifically, HDGF populations had greater fecundity at non-stressful temperatures and improved starvation tolerance at low temperatures (figure 3*A*,*B*), which are both consistent with adaptive life history responses [44,46]. As in the comparison of resistant and control populations, both gene flow populations showed improved cold tolerance when exposed to spinosad and NGF populations exhibited slower, putatively maladaptive recovery time on control medium (figure 3*C*). Intriguingly, the shifts in plasticity across temperature for fecundity and starvation tolerance occurred only when populations were exposed to spinosad during development. The induced response to temperature for NGF populations was weaker (in the case of fecundity) or absent (in the case of starvation tolerance). Although any magnified effect of both treatments was alleviated in the HDGF population for these phenotypes, this was not the case for body size or resistance (figure 3*D*; supplementary material, figure S4). Here, both populations exhibited similar plastic responses, with reduced body size and survival on spinosad at low temperature, evocative of the negative synergism found in the comparison of control and resistant populations.

Why did lower diversity result in the evolution of putatively maladaptive plasticity? Given reduced standing genetic variation relative to HDGF populations, the chances of matching phenotypic optima across temperatures and medium treatment were lower. This reinforces that rapid evolution of plasticity may largely be governed by similar processes to rapid evolution in other traits [12], although investigations of rates of plasticity evolution—relative to rates of mean shifts in traits—could reveal more complex patterns worthy of additional study. Broadly, expanding an understanding of the relationship between genetic diversity, the rate of plasticity evolution and the interaction between rapid trait evolution and plasticity evolution are critical to projecting organismal and population responses to environmental change.

### (c) Implications for the evolution of plasticity in multiple environments

Insecticides can be strongly deleterious to key insect pollinators [86] and can interact with thermal physiology [87,88]. In our study, when both insecticide and low temperature were experienced during development, all populations showed a strong decline in survival. This is important because effects in only one environment (e.g. insecticide exposure in warm temperature) might hide potentially negative outcomes that are only elicited when experiencing a different environment (exposure in low temperatures). Such ‘cryptic phenotypic plasticity’ has been suggested as a potential source of variation for selection to act on and indeed it could impact fecundity and vital rates [89,90]. However, as we found, maladaptive effects to plasticity could also be exacerbated under differing thermal regimes and could in part explain why resistance might trade-off with overwintering success [34,91]. Although this is potentially good news for managing agricultural pests, it is equally concerning for beneficial insects.

### (d) Limitations and future directions

There are important limitations to our study that should be considered when interpreting the results. First, the parallel evolution of reaction norms that we found is strong evidence for the action of natural selection driving the evolution of plasticity. From our study design, however, we cannot distinguish if plasticity in the traits we measured was the true target of selection or whether parallel shifts were due to correlated responses (i.e. pleiotropy or linkage). Second, our design structure had only two end points of temperature and spinosad treatment [72,92]. This prevented us from detecting nonlinear shapes of reaction norms that might differ at greater or lower environmental values [20,93]. Third, our single sampling time point means that we had limited temporal resolution of the evolution of plasticity across the season. Lastly, we did not directly test for shifts at the genomic or transcriptomic level and so the mechanisms that underlie plasticity evolution remain untested. Future work can address each of these gaps by incorporating more than two environmental levels and sampling at multiple junctures to determine whether the speed, direction or magnitude of plasticity evolution varies over the growing season. Finally, a transcriptomics approach using RNAseq and candidate loci for resistance and low temperatures could reveal genomic mechanisms of plasticity evolution.

## 5. Conclusion

Strong selection from sudden environmental shifts can reduce genetic diversity and impact population recovery with the loss of potentially beneficial alleles, including those that are plastic [56,94]. As we show here, assisted gene flow can provide an influx of novel alleles, including those that contribute to adaptive plasticity, that temper the impact of prior selection [5,95]. Indeed, this type of genetic rescue has been at the heart of conservation efforts for threatened species such as tropical corals through assisted gene flow [16,96]. Thus, the evolution of plasticity through selection or gene flow could be a critical, but underappreciated, feature of evolutionary rescue (reviewed in [9,97]. The complex outcomes in multiple environments, however, underscore the need for further investigation into how plasticity evolves in a changing world.

## Supplementary Material



## Data Availability

Data and code scripts available on the Open Science Framework [98]. Supplementary material is available online [99].
