# Supplementary material for: Insecticide resistance evolution and assisted gene flow interact to shape the evolution of plasticity
Source: Proc Biol Sci. 2026 Nov 11;293(2082):20262085. doi: 10.1098/rspb.2026.2085 (PMC13751116; doi:10.1098/rspb.2026.2085)
Supplement: Supplementary file 1 [file rspb.2026.2085.zip › rspb.2026.2085/rspb20262085_si_001.docx]

**Supplementary material for the article:**

**Title:**

Insecticide resistance evolution and assisted gene flow interact to shape the evolution of plasticity

**Authors**

Eric G. Prileson, Bianca Campagnari, Brynne Ruotsalainen, René S. Shahmohamadloo, Cristian Zetina, and Seth M. Rudman

Proceedings of the Royal Society B. doi: 10.1098/rspb.2016.2085

**Supplementary material**

*Details on phenotypic measurements*

Spinosad resistance

As a measure of resistance to the organic insecticide spinosad [Entrust; Dow AgroSciences, Indianapolis, IN, USA], we measured the successful development and survival of eggs to adults from each cage reared on both control and insecticide media and at the two test temperatures of 18 and 25 °C. Insecticide media contained 0.0375 ug L^-1^ spinosad concentration which was identified as the LC50 in the founder population using a standard toxicity assay and the same concentration used from the field experiment. We first encouraged flies to lay eggs on agar media with yeast in the common garden conditions 12 hours before collection. We placed 30 eggs into each replicate vial containing either spinosad or control media using a paintbrush. We then monitored development of all replicates and treatments every 24 hours until all flies had eclosed, at which point we counted the total number of surviving individuals as our measure of resistance.

Fecundity

We conducted fecundity assays with five females per replicate and three replicates per population following Rudman et al. (2022). Briefly, female flies were sorted under light anesthetic with CO_2_ and placed into flipped 50 mL bottles with food media as a lid. We then collected petri dishes from each bottle every 24 hours for three days and used a machine learning program (MLDAPP; Gabidulin and Rudman, 2023) to count eggs from photographs of each replicate dish for each day. We averaged the total number of eggs per female across the three day assay per population type and compared each population across both temperature and media treatments.

Starvation tolerance

We tested fly starvation tolerance by sorting 10 adult males per replicate and in three replicates per cage and allowed flies to recover for 30 minutes before starting the initial assay time. In this assay, we transferred 10 adult males into vials with agar but no food media and counted surviving individuals every 12 hours until all individuals had died following Rudman et al. (2022). Males were used for this assay to prevent laying behavior of females in the agar and metabolic loss from oogenesis (Markow and O’Grady, 2008)

Chill coma recovery time

To test cold tolerance of overwintered flies, we tested fly chill coma recovery time (CCRT; Gibert and Huey, 2001; Macdonald, 2004) with 15 female flies per cage with three replicates per cage. We based the chill coma temperature and time in chill coma from experimental data following Macdonald et al. (2004) and Rako and Hoffman (2006). Only one sex was used as there is strong female bias in body size, CCRT is strongly correlated with body size, and females are frequently used for CCRT trials (Gibert and Huey, 2001; Hallas et al. 2002, Rako and Hoffman, 2006). We sorted flies by sex using light CO_2_ anesthetic and placed anesthetized flies upside down into vials with food media and allowed flies to recover for two hours prior to the CCRT trial. Flies were placed upside down so they could recover on a foam cap and not become stuck in the media. To induce chill coma, we flipped vials right-side up and placed vials at 4 °C for a 10 hour period. We then monitored individual flies recovery at room temperature (20 °C) using a digital camera and video until all flies had recovered or until 30 minutes had elapsed. We scored recovery as the time to the second when flies were able to right themselves.

Dry mass

Following the fecundity assay, we measured the dry mass of female flies as a proxy for body size. To obtain the dry mass of each individual, we placed euthanized flies from each replicate into 1.5 mL tubes and placed the tubes within an incubator at 55 °C for 24 hours. We then weighed the mass of the flies using an analytical microbalance (Ohaus Pioneer PX) to the hundredth of a milligram ± 0.01 mg.

*Details on statistical analyses*

For all models, a significant effect of population type, treatment condition (temperature and/or media treatment), or their interaction would indicate evolution between populations, plasticity, or the evolution of plasticity, respectively. We constructed all models with the *glmmTMB()* function from the *glmmTMB* package (Brooks et al. 2017).

Question 1: Insecticide resistance and plasticity

Spinosad resistance:

To test the evolution of plasticity for resistance, we measured the mean survival among populations raised on spinosad and control media and at both 18 and 25 °C. For this model, we used percent survivorship as the response variable with population, temperature, and media treatment (along with their pairwise interactions) as our predictor variables, and cage as a random intercept. This model was constructed with a gaussian error distribution and the identity link function.

Starvation tolerance:

To test the evolution of plasticity for starvation tolerance across temperature, we measured the average time of survival in the absence of food among populations raised on control media and at both 18 and 25 °C. Separately, to test the evolution of plasticity for starvation tolerance across media treatment, we measured the time of survival in the absence of food among populations raised at 25 °C across both control and spinosad media. For the model across temperature, we used average hour of survival as the response variable with population, temperature, and their interaction as the predictor variables, and cage as a random intercept. Similarly, the model across media treatment also had average hour of survival as the response variable but with population, media treatment and their interaction as the predictor variables with cage as the random intercept. Both models were constructed with a gaussian error distribution and the identity link function.

Body size:

To test the evolution of plasticity for body size across temperature, we measured the average dry mass among populations raised on control media and at both 18 and 25 °C. Separately, to test the evolution of plasticity for body size across media treatment, we measured the average dry mass among populations raised at 25 °C across both control and spinosad media. For the model across temperature, we used average dry mass as the response variable with population, temperature, and their interaction as the predictor variables, and cage as a random intercept. Similarly, the model across media treatment also had average dry mass as the response variable but with population, media treatment and their interaction as the predictor variables with cage as the random intercept. Both models were constructed with a gaussian error distribution and the identity link function.

Fecundity:

To test the evolution of plasticity for fecundity across temperature, we measured the average eggs laid per female per day (over 3 days) among populations raised on control media and at both 18 and 25 °C. Separately, to test the evolution of plasticity for fecundity across media treatment, we measured the average eggs laid per female per day among populations raised at 25 °C across both control and spinosad media. For the model across temperature, we used average eggs laid per female per day (over 3 days) as the response variable with population, temperature, and their interaction as the predictor variables, and cage as a random intercept. Similarly, the model across media treatment also had average eggs laid per female per day (over 3 days) as the response variable but with population, media treatment and their interaction as the predictor variables with cage as the random intercept. Both models were constructed with a gaussian error distribution and the identity link function.

Chill coma recovery time:

To test the evolution of plasticity for cold tolerance across media treatment, we measured the average chill coma recovery time among populations raised at 25 °C across both control and spinosad media. For the model across media treatment, recovery time was the response variable with population, media treatment and their interaction as the predictor variables with cage as the random intercept. These models were constructed with a poisson error distribution and the log-link function.

Question 2: High diversity gene flow and plasticity

Fecundity:

To test the evolution of plasticity for fecundity, we measured the average eggs laid per female per day (over three days) among populations raised on spinosad and control media and at both 18 and 25 °C. For this model, we used average eggs per female per day as the response variable with population, temperature, and media treatment (along with their pairwise interactions) as our predictor variables, and cage as a random intercept. This model was constructed with a gaussian error distribution and the identity link function.

Starvation tolerance:

To test the evolution of plasticity for starvation tolerance, we measured the average hour of mortality in the absence of food among populations raised on spinosad and control media and at both 18 and 25 °C. For this model, we used average hour of death as the response variable with population, temperature, and media treatment (along with their pairwise interactions) as our predictor variables, and cage as a random intercept. This model was constructed with a gaussian error distribution and the identity link function.

Chill coma recovery time:

To test the evolution of plasticity for cold tolerance, we measured the time to recovery among populations raised on spinosad and control media and at 25 °C. For this model, we used recovery time as the response variable with population, temperature, and media treatment (along with their pairwise interactions) as our predictor variables, and cage as a random intercept. This model was constructed with a gaussian error distribution and the identity link function.

Body size:

To test the evolution of plasticity for body size, we measured the average dry mass among populations raised on spinosad and control media and at both 18 and 25 °C. For this model, we used average dry mass as the response variable with population, temperature, and media treatment (along with their pairwise interactions) as our predictor variables, and cage as a random intercept. This model was constructed with a gaussian error distribution and the identity link function.

Spinosad resistance:

To test the evolution of plasticity for resistance, we measured the mean survival among populations raised on spinosad and control media and at both 18 and 25 °C. For this model, we used percent survivorship as the response variable with population, temperature, and media treatment (along with their pairwise interactions) as our predictor variables, and cage as a random intercept. This model was constructed with a gaussian error distribution and the identity link function.

Figures

*Details on experimental mesocosms*

Figure S1. Schematic and timeline of the field study and laboratory experiment. (Left) Diagram and timeline of the multi-generational outdoor evolution experiment, showing each population type, the selection event from insecticide exposure, and gene flow treatment (see main text and supplementary information for details). (Right) Panels show steps of the common garden rearing, rearing treatment conditions, and phenotypes measured.

Figure S2. Fecundity (average eggs laid per female per day, for a 3 day assay) on control media measured across temperature for control (purple) and resistant (orange) populations. Large points show the mean trait value of each population type ± 1 SE. Smaller points jittered behind the means show raw data values for each independent population’s phenotypic measure.

Figure S3. Fecundity (average eggs laid per female per day, for a 3 day assay) measured across media treatment at 25 °C for control (purple) and resistant (orange) populations. Large points show the mean trait value of each population type ± 1 SE. Smaller points jittered behind the means show raw data values for each independent population’s phenotypic measure.

Figure S4. Spinosad resistance measured as the percent egg-to-adult survival on insecticide and control media across temperature (note: inset panels denote media treatments) for NGF (blue) and HDGF (gold) populations. Large points show the mean trait value of each population type ± 1 SE. Smaller points jittered behind the means show raw data values for each independent population’s phenotypic measure.

Tables

Table S1.

| **Date** | **Major category** | **Event** | **Steps** |
| --- | --- | --- | --- |
|  | Outdoor multigenerational experiment |  |  |
| 06/19/2024 |  | Outdoor introduction to mesocosms | Introduced all 44 populations (at this point both from 15 and 100 DGRP lines) into the orchard |
| 07/05/2024 |  | Spinosad insecticide treatments initiated | Replaced food media with spinosad treated food (0.0375 ug/L) |
| 08/5 – 08/16/2024 |  | Gene flow treatments initiated | Replaced original eggs laid with addition of 33% of population size with eggs from the HD control (100 DGRP outbred population) on 6 instances over 11 days |
| 10/6/2024 |  | Fall timepoint eggs collected | 800 eggs per cage in density controlled bottles from each population type |
| 10/24/2024 |  | Secondary fall time point eggs collected | A secondary time point was needed as some of the Fall time point cages went extinct in the laboratory following collection |
|  | Common Garden |  |  |
| 10/24 - 12/22/2024 |  | Indoor common garden rearing for 3 generations (P0, F1, F2) | 2 generations of common garden rearing at 25 C, 40% humidity, and a 12:12 LD light cycle |
| 12/12/2024 |  | Transfer of eggs laid by F2 adults to the four treatments | Density controlled eggs (30 eggs / vial) to either low temperature control, low temperature spinosad, warm temperature control, and warm temperature spinosad |
|  | Round 1 Phenotyping (25 C) |  |  |
| 1/2/2025 |  | Measurement of resistance | Measured survival / successful development on each treatment |
| 1/9/2025 |  | Fecundity day 1 and starvation tolerance | First day of fecundity measures and beginning of the starvation tolerance assay |
| 1/10/2025 |  | Fecundity day 2 and CCRT | Second day of fecundity and cold tolerance assay |
| 1/11/2025 |  | Fecundity day 3 and end of starvation tolerance | Last day of fecundity measure and starvation tolerance end point |
| 1/13/2025 |  | Dry mass measure | Desiccation for 12 hours |
|  | Round 2 Phenotyping (18 C) |  |  |
| 1/15/2025 |  | Measurement of resistance | Measured survival / successful development on each treatment |
| 1/16/2025 |  | Fecundity day 1 and starvation tolerance | First day of fecundity measures and beginning of the starvation tolerance assay |
| 1/17/2025 |  | Fecundity day 2 and CCRT | Second day of fecundity and cold tolerance assay |
| 1/18/2025 |  | Fecundity day 3 and end of starvation tolerance | Last day of fecundity measure and starvation tolerance end point |
| 1/20/2025 |  | Dry mass measure | Desiccation for 12 hours |

Table S2. Proportion of flies reared at 18 C that did not undergo chill coma

| **Cage** | **Type** | **# flies in chill coma** | **Total flies** | **Percent not in chill coma** | **Treatment** |
| --- | --- | --- | --- | --- | --- |
| 2 | Control | 4 | 15 | 0.73 | Control |
| 4 | Resistant | 5 | 13 | 0.62 | Control |
| 5 | Control | 2 | 15 | 0.87 | Control |
| 6 | Resistant | 4 | 15 | 0.73 | Control |
| 8 | Control | 4 | 15 | 0.73 | Control |
| 9 | Resistant | 3 | 15 | 0.8 | Control |
| 10 | Control | 4 | 15 | 0.73 | Control |
| 11 | Resistant | 2 | 15 | 0.87 | Control |
| 11 | Resistant | 1 | 15 | 0.93 | Spinosad |
| 12 | Resistant | 4 | 15 | 0.73 | Control |
| 13 | Control | 2 | 15 | 0.87 | Control |
| 15 | Control | 5 | 15 | 0.67 | Control |
| 19 | Resistant | 2 | 15 | 0.87 | Control |
| 19 | Resistant | 0 | 12 | 0 | Spinosad |
| 20 | Resistant | 3 | 15 | 0.8 | Control |
| 22 | Control | 3 | 15 | 0.8 | Control |
| 23 | Control | 2 | 15 | 0.87 | Control |
| 27 | Control | 2 | 15 | 0.87 | Control |
| 30 | Resistant | 2 | 15 | 0.87 | Control |
| 31 | Resistant | 1 | 15 | 0.93 | Control |
| 32 | Control | 4 | 15 | 0.73 | Control |
| 33 | Resistant | 2 | 15 | 0.87 | Control |
| 34 | Control | 2 | 15 | 0.87 | Control |
| 36 | Resistant | 3 | 15 | 0.8 | Control |
| 41 | Control | 1 | 15 | 0.93 | Control |
| 42 | Control | 2 | 15 | 0.87 | Control |
| 43 | Resistant | 4 | 15 | 0.73 | Control |
| 44 | Resistant | 1 | 15 | 0.93 | Control |
| 50 | Control | 1 | 15 | 0.93 | Control |
| 54 | Resistant | 1 | 15 | 0.93 | Control |
| 60 | Resistant | 4 | 15 | 0.73 | Control |

Table S3.  Predicted outcomes of reaction norms for measured phenotypes in response to temperature that are consistent with adaptation based on prior literature.

| **Phenotype** | **Temperature** | **Optimal reaction norm direction (slope)** | References |
| --- | --- | --- | --- |
| Body size | Low to High | Decreasing (negative) | Stevenson, 1985; Partridge et al. 1994; Reznick et al. 2000 |
| Fecundity | Low to High | Increasing (positive) | Schmidt et al. 2005; Markow and O’Grady, 2008; Schmidt and Paaby, 2008 |
| Starvation tolerance | Low to High | Decreasing (negative) | Rion and Kawecki, 2007; Rudman et al. 2022 |
| Survival | Low to High | Flat (zero) | McKenzie et al. 1990; Yang et al. 2018; Engell Dahl et al. 2021 |

Table S4. Proportion survival of flies within each temperature and media treatment.

| **Population type** | **Treatment** | **Temperature** | **Survival_percent** | **SE** |
| --- | --- | --- | --- | --- |
| Control | Control | 18 | 88 | 3.02 |
| Control | Spinosad | 18 | 1.39 | 0.645 |
| Resistant | Control | 18 | 84.3 | 3.36 |
| Resistant | Spinosad | 18 | 24.5 | 3.37 |
| Control | Control | 25 | 88.2 | 5.42 |
| Control | Spinosad | 25 | 33.1 | 3.11 |
| Resistant | Control | 25 | 85.8 | 2.99 |
| Resistant | Spinosad | 25 | 74.3 | 4.23 |
| HD gene flow | Control | 18 | 90.8 | 4.4 |
| HD gene flow | Spinosad | 18 | 20.4 | 4.84 |
| No gene flow | Control | 18 | 83.4 | 4.14 |
| No gene flow | Spinosad | 18 | 15.2 | 2.92 |
| HD gene flow | Control | 25 | 87.3 | 4.47 |
| HD gene flow | Spinosad | 25 | 72.8 | 6.14 |
| No gene flow | Control | 25 | 86.8 | 3.92 |
| No gene flow | Spinosad | 25 | 66.2 | 5.14 |

Table S5. Levene’s test for differences in variance of reaction norm slopes between populations

| **Population comparison** | **Phenotype** | **F** | **P** |
| --- | --- | --- | --- |
| Resistant vs. control | Body size (across temperature) | 0.556 | 0.460 |
|  | Body size (across media treatment) | 0.0863 | 0.770 |
|  | Fecundity (across temperature) | 0.270 | 0.604 |
|  | Fecundity (across media treatment) | 1.83 | 0.179 |
|  | Starvation tolerance (across temperature) | 2.91 | 0.0941 |
|  | Starvation tolerance (across media treatment) | 0.306 | 0.583 |
|  | Resistance | 1.04 | 0.375 |
|  | Chill coma recovery time (across media treatment) | 3.70 | 0.0562 |
|  |  |  |  |
| NGF vs. HDGF | Body size | 1.09 | 0.368 |
|  | Fecundity | 0.207 | 0.891 |
|  | Starvation tolerance | 1.44 | 0.250 |
|  | Resistance | 0.678 | 0.568 |
|  | Chill coma recovery time | 0.832 | 0.365 |

Table S6. Model estimates and outputs for each phenotype for the first question of insecticide resistance and the evolution of plasticity.

| **Phenotypic measure** | **Term** | **Estimate** | **SE** | **Χ^2^** | **P** |
| --- | --- | --- | --- | --- | --- |
| Resistance |  |  |  |  |  |
|  | Population | -4.22 | 3.30 | 1.63 | 0.201 |
|  | Temperature | -1.34 | 2.98 | 0.203 | 0.652 |
|  | Treatment | -86.3 | 2.98 | 840.5 | **< 0.0001** |
|  | Temperature : Population | 3.45 | 3.81 | 0.821 | 0.365 |
|  | Media Treatment : Pop | 27.4 | 3.81 | 51.6 | **< 0.0001** |
|  | Temperature : Treatment | 33.1 | 4.21 | 61.8 | **< 0.0001** |
|  | Population : Temperature : Media Treatment | 14.4 | 5.39 | 7.15 | **0.00751** |
| Starvation tolerance (Temperature; control media) |  |  |  |  |  |
|  | Population | -6.26 | 3.06 | 4.18 | **0.0409** |
|  | Temperature | -6.57 | 2.23 | 8.71 | **0.00316** |
|  | Population : Temperature | 5.66 | 2.85 | 3.95 | **0.0468** |
|  |  |  |  |  |  |
| Starvation tolerance (media treatment 25 °C) |  |  |  |  |  |
|  | Population | -0.597 | 2.64 | 0.0510 | 0.821 |
|  | Media treatment | -10.3 | 2.92 | 12.4 | **0.000435** |
|  | Population : Media treatment | 10.1 | 3.74 | 7.30 | **0.00690** |
| Chill coma recovery time (media treatment 25 °C) |  |  |  |  |  |
|  | Population | 7.06 | 1.15 | 3.91 | **0.0480** |
|  | Media treatment | -6.95 | 1.15 | 8.06 | **0.00452** |
|  | Population : media treatment | -8.17 | 1.19 | 7.88 | **0.00500** |
| Dry mass (Temperature; control media) |  |  |  |  |  |
|  | Population | -0.00831 | 0.0597 | 0.00190 | 0.908 |
|  | Temperature | -0.194 | 0.0715 | 22.6 | **< 0.0001** |
|  | Population : Temperature | -0.12 | 0.0912 | 12.4 | **0.000428** |
| Dry mass (media treatment; 25 °C) |  |  |  |  |  |
|  | Population | -0.138 | 0.064 | 8.03 | **0.00460** |
|  | Media treatment | 0.16 | 0.0718 | 13.4 | **0.000253** |
|  | Population : media treatment | 0.178 | 0.0909 | 14.3 | **0.000154** |
| Fecundity (Temperature; control media) |  |  |  |  |  |
|  | Population | -0.630 | 1.88 | 0.113 | 0.737 |
|  | Temperature | 18.2 | 1.90 | 92.2 | **< 0.0001** |
|  | Population : Temperature | -0.0243 | 2.43 | 0.000100 | 0.992 |
| Fecundity (media treatment; 25 °C) |  |  |  |  |  |
|  | Population | -0.654 | 2.06 | 0.101 | 0.751 |
|  | Media treatment | -7.66 | 1.83 | 17.4 | **< 0.0001** |
|  | Population : media treatment | 4.13 | 2.35 | 3.10 | 0.0785 |

Table S7. Estimated marginal means and contrasts between levels for predictor variables for insecticide resistance and evolution of plasticity. Lower and upper confidence levels represent 95 % confidence intervals.

| **Phenotype** | **Population** | **Temperature** | **Media Treatment** | **Estimated marginal mean** | **SE** | **df** | **Lower CL** | **Upper CL** |
| --- | --- | --- | --- | --- | --- | --- | --- | --- |
| Resistance |  |  |  |  |  |  |  |  |
|  | Control | 18 | Control | 88 | 2.52 | Inf | 83 | 92.9 |
|  | Resistant | 18 | Control | 84.3 | 2.01 | Inf | 80.4 | 88.2 |
|  | Control | 25 | Control | 88.2 | 2.52 | Inf | 83.2 | 93.1 |
|  | Resistant | 25 | Control | 85.8 | 2.01 | Inf | 81.9 | 89.8 |
|  | Control | 18 | Spinosad | 1.39 | 2.28 | Inf | -3.08 | 5.86 |
|  | Resistant | 18 | Spinosad | 24.5 | 1.82 | Inf | 21 | 28.1 |
|  | Control | 25 | Spinosad | 33.1 | 2.28 | Inf | 28.7 | 37.6 |
|  | Resistant | 25 | Spinosad | 74.3 | 1.82 | Inf | 70.8 | 77.9 |
|  |  |  |  |  |  |  |  |  |
|  | **Population Contrast** | **Temperature** | **Media Treatment** | **estimate** | **SE** | **df** | **Z.ratio** | **P** |
|  |  |  |  |  |  |  |  |  |
|  | Control - Resistant | 18 | Control | 4.22 | 3.30 | Inf | 1.23 | 0.201 |
|  | Control - Resistant | 25 | Control | 0.771 | 3.30 | Inf | 0.234 | 0.815 |
|  | Control - Resistant | 18 | Spinosad | -23.1 | 3.30 | Inf | -7.001 | **< 0.0001** |
|  | Control - Resistant | 25 | Spinosad | -41.0 | 3.30 | Inf | -12.4 | **< 0.0001** |
| Starvation (temperature; control media) |  |  |  |  |  |  |  |  |
|  | **Population** | **Temperature** | **Estimated marginal mean** | **SE** | **df** | **Lower CL** | **Upper CL** |  |
|  |  |  |  |  |  |  |  |  |
|  | Control | 18 | 83.5 | 2.3 | 17.8 | 78.7 | 88.3 |  |
|  | Control | 25 | 77.1 | 2.29 | 17.6 | 72.3 | 82 |  |
|  | Resistant | 18 | 77.5 | 1.83 | 17.6 | 73.6 | 81.3 |  |
|  | Resistant | 25 | 76.6 | 1.83 | 17.8 | 72.7 | 80.4 |  |
|  |  |  |  |  |  |  |  |  |
|  | **Contrasts** | **Temperature** | **estimate** | **SE** | **df** | **Z.ratio** | **P** |  |
|  | Control - Resistant | 18 °C | 6.26 | 3.06 | Inf | 2.04 | **0.0409** |  |
|  | Control - Resistant | 25 °C | 0.597 | 3.06 | Inf | 0.195 | 0.845 |  |
| Starvation (Media treatment; 25 °C) |  |  |  |  |  |  |  |  |
|  | **Population** | **Media Treatment** | **Estimated marginal mean** | **SE** | **df** | **Lower CL** | **Upper CL** |  |
|  | Control | Control | 77.1 | 1.4 | 31 | 74.3 | 80 |  |
|  | Control | Spinosad | 66.6 | 1.55 | 41.2 | 63.5 | 69.7 |  |
|  | Resistant | Control | 76.8 | 1.13 | 32 | 74.5 | 79.1 |  |
|  | Resistant | Spinosad | 76.4 | 1.12 | 31 | 74.1 | 78.6 |  |
|  |  |  |  |  |  |  |  |  |
|  | **Contrasts** | **Treatment** | **estimate** | **SE** | **df** | **Z.ratio** | **P** |  |
|  | Control - Resistant | Control | 0.597 | 2.64 | Inf | 0.226 | 0.821 |  |
|  | Control - Resistant | Spinosad | -9.51 | 2.64 | Inf | -3.60 | **0.000300** |  |
| Chill coma recovery time (Media treatment; 25 °C) |  |  |  |  |  |  |  |  |
|  | **Population** | **Media Treatment** | **Estimated marginal mean** | **SE** | **df** | **Lower CL** | **Upper CL** |  |
|  | Control | Control | 21.4 | 2.53 | Inf | 16.4 | 26.3 |  |
|  | Resistant | Control | 28.4 | 2.31 | Inf | 23.9 | 32.9 |  |
|  | Control | Spinosad | 14.4 | 1.78 | Inf | 10.9 | 17.9 |  |
|  | Resistant | Spinosad | 11.8 | 1.24 | Inf | 9.37 | 14.2 |  |
|  |  |  |  |  |  |  |  |  |
|  | **Contrasts** | **Treatment** | **estimate** | **SE** | **df** | **Z.ratio** | **P** |  |
|  | Control - Resistant | Control | -7.06 | 3.44 | Inf | -2.05 | **0.0403** |  |
|  | Control - Resistant | Spinosad | 2.61 | 2.16 | Inf | 1.21 | 0.227 |  |
|  |  |  |  |  |  |  |  |  |
| Dry mass (temperature; control media) |  |  |  |  |  |  |  |  |
|  | **Population** | **Temperature** | **Estimated marginal mean** | **SE** | **df** | **Lower CL** | **Upper CL** |  |
|  | Control | 18 | 2.43 | 0.043 | Inf | 2.34 | 2.51 |  |
|  | Control | 25 | 2.28 | 0.042 | Inf | 2.2 | 2.37 |  |
|  | Resistant | 18 | 2.42 | 0.034 | Inf | 2.36 | 2.49 |  |
|  | Resistant | 25 | 2.14 | 0.034 | Inf | 2.08 | 2.21 |  |
|  |  |  |  |  |  |  |  |  |
|  | **Contrasts** | **Temperature** | **estimate** | **SE** | **df** | **Z.ratio** | **P** |  |
|  | Control – Resistant | 18 | 0.00626 | 0.0543 | Inf | 0.115 | 0.908 |  |
|  | Control – Resistant | 25 | 0.142 | 0.0540 | Inf | 2.63 | **0.00860** |  |
| Dry mass (media treatment; 25 °C) |  |  |  |  |  |  |  |  |
|  | **Population** | **Media Treatment** | **Estimated marginal mean** | **SE** | **df** | **Lower CL** | **Upper CL** |  |
|  | Control | Control | 2.28 | 0.039 | Inf | 2.21 | 2.36 |  |
|  | Resistant | Control | 2.14 | 0.031 | Inf | 2.08 | 2.2 |  |
|  | Control | Spinosad | 2.43 | 0.039 | Inf | 2.35 | 2.51 |  |
|  | Resistant | Spinosad | 2.48 | 0.031 | Inf | 2.42 | 2.54 |  |
|  |  |  |  |  |  |  |  |  |
|  | **Contrasts** | **Media Treatment** | **estimate** | **SE** | **df** | **Z.ratio** | **P** |  |
|  | Control - Resistant | Control | 0.142 | 0.05 | Inf | 2.83 | **0.00460** |  |
|  | Control - Resistant | Spinosad | -0.05 | 0.05 | Inf | -0.998 | 0.318 |  |
| Fecundity (Temperature; control media) |  |  |  |  |  |  |  |  |
|  | **Population** | **Temperature** | **Estimated marginal mean** | **SE** | **df** | **Lower CL** | **Upper CL** |  |
|  | 18 | Control | 16.5 | 0.942 | Inf | 14.6 | 18.3 |  |
|  | 25 | Control | 34.7 | 1.37 | Inf | 32 | 37.4 |  |
|  | 18 | Resistant | 15.8 | 0.736 | Inf | 14.4 | 17.3 |  |
|  | 25 | Resistant | 34 | 1.08 | Inf | 31.9 | 36.1 |  |
|  |  |  |  |  |  |  |  |  |
|  | **Contrasts** | **Population** | **estimate** | **SE** | **df** | **Z.ratio** | **P** |  |
|  | Control - Resistant | 18 | 0.630 | 1.88 | Inf | 0.335 | 0.737 |  |
|  | Control - Resistant | 25 | 0.654 | 1.88 | Inf | 0.348 | 0.728 |  |
| Fecundity (media treatment; 25 °C) |  |  |  |  |  |  |  |  |
|  | **Population** | **Media Treatment** | **Estimated marginal mean** | **SE** | **df** | **Lower CL** | **Upper CL** |  |
|  | Control | Control | 34.8 | 1.34 | Inf | 32.2 | 37.5 |  |
|  | Resistant | Control | 34.2 | 1.07 | Inf | 32.1 | 36.3 |  |
|  | Control | Spinosad | 27.2 | 1.34 | Inf | 24.6 | 29.8 |  |
|  | Resistant | Spinosad | 30.7 | 1.07 | Inf | 28.6 | 32.8 |  |
|  |  |  |  |  |  |  |  |  |
|  | **Contrasts** | **Media Treatment** | **estimate** | **SE** | **df** | **Z.ratio** | **P** |  |
|  | Control - Resistant | Control | 0.654 | 2.06 | Inf | 0.318 | 0.751 |  |
|  | Control - Resistant | Spinosad | -3.47 | 2.06 | Inf | -1.69 | 0.0918 |  |

Table S8. Model estimates and outputs for each phenotype for the second question of gene flow and the evolution of plasticity.

| **Phenotypic measure** | **Term** | **Levels** | **Estimate** | **SE** | **Χ^2^** | **P** |
| --- | --- | --- | --- | --- | --- | --- |
| Fecundity | Population |  | 1.57 | 2.08 | 0.573 | 0.449 |
|  | Media Treatment |  | 1.4 | 0.735 | 3.64 | 0.0565 |
|  | Temperature |  | 18.7 | 0.653 | 821.7 | **< 0.0001** |
|  | Population : Media Treament |  | -1.06 | 2.62 | 0.163 | 0.687 |
|  | Population : Temperature |  | -2.53 | 2.62 | 0.934 | 0.334 |
|  | Media Treatment : Temperature |  | -8.83 | 2.62 | 11.4 | **0.000752** |
|  | Population : Media Treatment :Temperature |  | 7.49 | 3.71 | 4.09 | **0.0409** |
| Starvation tolerance |  |  |  |  |  |  |
|  | Population |  | 6.000 | 3.66 | 2.69 | 0.101 |
|  | Media Treatment |  | -1.50 | 3.66 | 0.168 | 0.682 |
|  | Temperature |  | -1.50 | 3.66 | 0.168 | 0.681 |
|  | Population : Media Treament |  | 4.000 | 5.18 | 0.597 | 0.440 |
|  | Population : Temperature |  | -1.50 | 5.18 | 0.0840 | 0.772 |
|  | Media Treatment : Temperature |  | 5.000 | 5.18 | 0.934 | 0.334 |
|  | Population : Media Treatment :Temperature |  | -12.5 | 7.32 | 2.92 | 0.0876 |
| CCRT |  |  |  |  |  |  |
|  | Population |  | -10.0 | 2.44 | 16.9 | **< 0.0001** |
|  | Treatment |  | -17.3 | 0.920 | 351.2 | **< 0.0001** |
|  | Pop*Treatment |  | 8.78 | 1.40 | 39.3 | **< 0.0001** |
| Resistance |  |  |  |  |  |  |
|  | Population |  | 6.65 | 5.08 | 1.72 | 0.190 |
|  | Treatment |  | -67.4 | 3.43 | 385.6 | **< 0.0001** |
|  | Temperature |  | 3.70 | 3.43 | 1.16 | 0.281 |
|  | Pop*Treatment |  | -1.49 | 4.85 | 0.0948 | 0.758 |
|  | Pop*Temp |  | -7.000 | 4.85 | 2.08 | 0.149 |
|  | Treatment*Temp |  | 47.2 | 4.85 | 94.7 | **< 0.0001** |
|  | Pop*Treatment*Temp |  | 8.41 | 6.86 | 1.50 | 0.220 |

Table S9. Estimated marginal means and contrasts between levels for predictor variables for gene flow and the evolution of plasticity. Lower and upper confidence levels represent 95 % confidence intervals.

| **Phenotype** | **Population** | **Media Treatment** | **Temperature** | **Estimated marginal mean** | **SE** | **df** | **Lower CL** | **Upper CL** |
| --- | --- | --- | --- | --- | --- | --- | --- | --- |
| Fecundity | NGF | Control | 18 | 16 | 0.968 | Inf | 14.1 | 17.9 |
|  | HDGF | Control | 18 | 17.6 | 0.968 | Inf | 15.7 | 19.4 |
|  | NGF | Spinosad | 18 | 17.4 | 1 | Inf | 15.5 | 19.4 |
|  | HDGF | Spinosad | 18 | 17.6 | 0.975 | Inf | 15.7 | 19.6 |
|  | NGF | Control | 25 | 34.7 | 0.968 | Inf | 32.8 | 36.6 |
|  | HDGF | Control | 25 | 33.7 | 0.968 | Inf | 31.9 | 35.6 |
|  | NGF | Spinosad | 25 | 27.1 | 0.968 | Inf | 25.2 | 29 |
|  | HDGF | Spinosad | 25 | 32.6 | 0.968 | Inf | 30.7 | 34.5 |
|  |  |  |  |  |  |  |  |  |
|  | **Contrasts** |  |  |  |  |  |  |  |
|  | NGF - HDGF | Control | 18 | -1.57 | 1.37 | Inf | -1.15 | 0.251 |
|  | NGF - HDGF | Spinosad | 18 | -0.224 | 1.4 | Inf | -0.16 | 0.873 |
|  | NGF - HDGF | Control | 25 | 0.962 | 1.37 | Inf | 0.703 | 0.482 |
|  | NGF - HDGF | Spinosad | 25 | -5.47 | 2.08 | Inf | -2.64 | **< 0.00840** |
|  |  |  |  |  |  |  |  |  |
| Starvation tolerance | **Population** | **Media Treatment** | **Temperature** | **Estimated marginal mean** | **SE** | **df** | **Lower CL** | **Upper CL** |
|  | NGF | Control | 18 | 75 | 1.73 | 76 | 71.6 | 78.4 |
|  | HDGF | Control | 18 | 81 | 1.73 | 76 | 77.6 | 84.4 |
|  | NGF | Control | 25 | 74 | 1.78 | 76 | 70.5 | 77.6 |
|  | HDGF | Control | 25 | 78 | 1.73 | 76 | 74.6 | 81.4 |
|  | NGF | Spinosad | 18 | 73.7 | 2.26 | 76 | 69.2 | 78.2 |
|  | HDGF | Spinosad | 18 | 84.4 | 1.93 | 76 | 80.5 | 88.2 |
|  | NGF | Spinosad | 25 | 77 | 1.73 | 76 | 73.6 | 80.4 |
|  | HDGF | Spinosad | 25 | 73 | 1.73 | 76 | 69.6 | 76.4 |
|  |  |  |  |  |  |  |  |  |
|  | **Contrasts** | **Media Treatment** | **Temperature** | **estimate** | **SE** | **df** | **Z.ratio** | **P** |
|  | NGF - HDGF | Control | 18 | -6.000 | 3.66 | Inf | -1.64 | 0.101 |
|  | NGF - HDGF | Control | 25 | -3.99 | 2.48 | Inf | -1.61 | 0.112 |
|  | NGF - HDGF | Spinosad | 18 | -10.0 | 3.66 | Inf | -2.73 | **0.00630** |
|  | NGF - HDGF | Spinosad | 25 | 4 | 2.44 | Inf | 1.64 | 0.105 |
|  |  |  |  |  |  |  |  |  |
| Chill coma recovery time | **Population** | **Media Treatment** | **Estimated marginal mean** | **SE** | **df** | **Lower CL** | **Upper CL** |  |
|  | NGF | Control | 31.3 | 2.41 | 12.7 | 26 | 36.5 |  |
|  | HDGF | Control | 21.1 | 2.46 | 13.8 | 15.8 | 26.4 |  |
|  | No_gene_flow | Spinosad | 14 | 2.41 | 12.7 | 8.77 | 19.2 |  |
|  | HD_gene_flow | Spinosad | 12.8 | 2.41 | 12.7 | 7.52 | 18 |  |
|  | **Contrasts** | **Media Treatment** | **estimate** | **SE** | **df** | **Z.ratio** | **P** |  |
|  | NGF - HDGF | Control | 10.0 | 2.44 | Inf | 4.11 | **< 0.0001** |  |
|  | NGF - HDGF | Spinosad | 1.25 | 2.39 | Inf | 0.524 | 0.6000 |  |
|  |  |  |  |  |  |  |  |  |
| Dry mass | **Media Treatment** | **Population** | **Temperature** | **Estimated marginal mean** | **SE** | **df** | **Lower CL** | **Upper CL** |
|  | Control | NGF | 18 | 2.58 | 0.072 | 8.6 | 2.41 | 2.74 |
|  | Spinosad | NGF | 18 | 2.23 | 0.075 | 10.1 | 2.07 | 2.4 |
|  | Control | HDGF | 18 | 2.44 | 0.073 | 8.99 | 2.27 | 2.6 |
|  | Spinosad | HDGF | 18 | 2.22 | 0.073 | 8.99 | 2.05 | 2.38 |
|  | Control | NGF | 25 | 2.08 | 0.072 | 8.27 | 1.92 | 2.25 |
|  | Spinosad | NGF | 25 | 2.43 | 0.072 | 8.27 | 2.27 | 2.6 |
|  | Control | HDGF | 25 | 2.18 | 0.072 | 8.27 | 2.02 | 2.35 |
|  | Spinosad | HDGF | 25 | 2.51 | 0.072 | 8.27 | 2.35 | 2.68 |
|  |  |  |  |  |  |  |  |  |
|  | **Contrasts** | **Population** | **Temperature** | **estimate** | **SE** | **df** | **Z.ratio** | **P** |
|  | Control - Spinosad | NGF | 18 | 0.343 | 0.0506 | 73.1 | 6.77 | **< 0.0001** |
|  | Control - Spinosad | HDGF | 18 | 0.223 | 0.0487 | 73.1 | 4.58 | **< 0.0001** |
|  | Control - Spinosad | NGF | 25 | -0.353 | 0.0443 | 73 | -7.96 | **< 0.0001** |
|  | Control - Spinosad | HDGF | 25 | -0.330 | 0.0443 | 73 | -7.45 | **< 0.0001** |
|  |  |  |  |  |  |  |  |  |
| Resistance | **Temperature** | **Population** | **Media Treatment** | **Estimated marginal mean** | **SE** | **df** | **Lower CL** | **Upper CL** |
|  | 18 | NGF | Control | 83.5 | 3.67 | 27.9 | 75.9 | 91 |
|  | 25 | NGF | Control | 86.8 | 3.67 | 27.9 | 79.3 | 94.3 |
|  | 18 | HDGF | Control | 90.7 | 3.67 | 27.9 | 83.2 | 98.3 |
|  | 25 | HDGF | Control | 87.3 | 3.67 | 27.9 | 79.8 | 94.8 |
|  | 18 | NGF | Spinosad | 15.2 | 3.36 | 19.8 | 8.23 | 22.3 |
|  | 25 | NGF | Spinosad | 66.2 | 3.36 | 19.8 | 59.1 | 73.2 |
|  | 18 | HDGF | Spinosad | 20.4 | 3.36 | 19.8 | 13.4 | 27.4 |
|  | 25 | HDGF | Spinosad | 72.8 | 3.36 | 19.8 | 65.8 | 79.8 |
|  |  |  |  |  |  |  |  |  |
|  | **Contrasts** | **Temperature** | **Media Treatment** | **estimate** | **SE** | **df** | **Z.ratio** | **P** |
|  | NGF - HDGF | 18 | Control | -6.65 | 5.08 | Inf | -1.31 | 0.190 |
|  | NGF - HDGF | 25 | Control | 0.350 | 5.08 | Inf | 0.0690 | 0.945 |
|  | NGF - HDGF | 18 | Spinosad | -5.16 | 5.08 | Inf | -1.02 | 0.310 |
|  | NGF - HDGF | 25 | Spinosad | -6.56 | 5.08 | Inf | -1.29 | 0.196 |

Table S10. Comparison of high diversity control populations (HD_control) with high diversity gene flow populations (HDGF) to document how much of the phenotypic variation was due to the gene flow event. Statistics show main effect of population. If the main effect or interaction was significant, we also show tests for marginal means between populations among treatments. Note: since HD_control populations did not survive at sufficient levels on spinosad media, we test for differences in reaction norms on media treatment and temperature separately except for the resistance phenotype.

| **Phenotypic measure** | **Term** | **Estimate** | **SE** | **Χ^2^** | **P** |
| --- | --- | --- | --- | --- | --- |
| Resistance |  |  |  |  |  |
|  | Population | 0.525 | 1.92 | 0.0249 | 0.875 |
|  | Temperature | 0.325 | 0.934 | 0.121 | 0.728 |
|  | Treatment | -88.4 | 0.842 | 11036.9 | **< 0.0001** |
|  | Temperature : Population | -3.78 | 1.62 | 5.45 | **0.0196** |
|  | Media Treatment : Pop | 18.1 | 1.46 | 153.7 | **< 0.0001** |
|  | Temperature : Treatment | 31.6 | 3.14 | 796.9 | **< 0.0001** |
|  | Population : Temperature : Media Treatment | 16.7 | 4.02 | 116.6 | **< 0.0001** |
| Starvation tolerance (Temperature; control media) |  |  |  |  |  |
|  | Population | 6.00 | 2.90 | 4.27 | **0.0388** |
|  | Temperature | -1.52 | 1.55 | 0.967 | 0.326 |
|  | Population : Temperature | -1.48 | 2.63 | 0.315 | 0.574 |
|  |  |  |  |  |  |
| Starvation tolerance (media treatment 25 °C) |  |  |  |  |  |
|  | Population | 4.86 | 2.56 | 1.24 | 0.266 |
|  | Media treatment | -8.84 | 2.10 | 17.7 | **< 0.0001** |
|  | Population : Media treatment | 3.84 | 3.50 | 1.21 | 0.272 |
| Chill coma recovery time (media treatment 25 °C) |  |  |  |  |  |
|  | Population | -11.3 | 4.35 | 6.77 | **0.00929** |
|  | Media treatment | -16.5 | 0.650 | 165.4 | **< 0.0001** |
|  | Population : media treatment | 7.80 | 1.06 | 54.01 | **< 0.0001** |
| Dry mass (Temperature; control media) |  |  |  |  |  |
|  | Population | 0.154 | 0.104 | 2.22 | 0.136 |
|  | Temperature | -0.152 | 0.0501 | 9.27 | **0.00234** |
|  | Population : Temperature | -0.109 | 0.0875 | 1.55 | 0.213 |
| Dry mass (media treatment; 25 °C) |  |  |  |  |  |
|  | Population | 0.0453 | 0.0713 | 0.404 | 0.525 |
|  | Media treatment | 0.306 | 0.0321 | 91.3 | **< 0.0001** |
|  | Population : media treatment | 0.0239 | 0.0544 | 0.193 | 0.660 |
| Fecundity (Temperature; control media) |  |  |  |  |  |
|  | Population | 0.346 | 2.01 | 0.0295 | 0.864 |
|  | Temperature | 19.4 | 0.379 | 2623.8 | **< 0.0001** |
|  | Population : Temperature | -3.19 | 0.652 | 24.0 | **< 0.0001** |
| Fecundity (media treatment; 25 °C) |  |  |  |  |  |
|  | Population | -2.81 | 1.93 | 2.13 | 0.145 |
|  | Media treatment | -4.43 | 0.612 | 52.3 | **< 0.0001** |
|  | Population : media treatment | 3.27 | 1.06 | 9.60 | **0.00195** |

Table S11. Contrasts of estimated marginal mean differences in reaction norms between HD_control and HDGF populations. Contrasts are of phenotypes with significant terms of population type or interactions between population type, temperature, and/or media treatment from model outputs shown in Table S10.

| **Phenotype** | **Population Contrast** | **Temperature** | **Media Treatment** | **estimate** | **SE** | **df** | **Z.ratio** | **P** |
| --- | --- | --- | --- | --- | --- | --- | --- | --- |
| Resistance |  |  |  |  |  |  |  |  |
|  | HD_Control - HDGF | 18 | Control | -0.525 | 3.33 | Inf | -0.158 | 0.875 |
|  | HD_Control - HDGF | 25 | Control | 3.25 | 3.33 | Inf | 0.977 | 0.329 |
|  | HD_Control - HDGF | 18 | Spinosad | -18.6 | 3.25 | Inf | -5.72 | **< 0.0001** |
|  | HD_Control - HDGF | 25 | Spinosad | -37.1 | 3.25 | Inf | -11.4 | **< 0.0001** |
| Starvation (temperature; control media) |  |  |  |  |  |  |  |  |
|  | **Contrasts** | **Temperature** | **Media treatment** | **estimate** | **SE** | **df** | **Z.ratio** | **P** |
|  | HD_Control - HDGF | 18 | Control | -6.00 | 2.90 | Inf | -2.07 | **0.0388** |
|  | HD_Control - HDGF | 25 | Control | -4.52 | 2.93 | Inf | -1.55 | 0.122 |
| Chill coma recovery time (Media treatment; 25 °C) |  |  |  |  |  |  |  |  |
|  | **Contrasts** | **Temperature** | **Treatment** | **estimate** | **SE** | **df** | **t.ratio** | **P** |
|  | HD_Control - HDGF | 25 | Control | 11.3 | 4.35 | 9.42 | 2.60 | **0.0277** |
|  | HD_Control - HDGF | 25 | Spinosad | 3.53 | 4.32 | 9.14 | 0.816 | 0.435 |
| Fecundity (Temperature; control media) |  |  |  |  |  |  |  |  |
|  | **Contrasts** | **Temperature** | **Treatment** | **estimate** | **SE** | **df** | **Z.ratio** | **P** |
|  | HD_Control - HDGF | 18 | Control | -0.346 | 2.01 | Inf | -0.172 | 0.864 |
|  | HD_Control - HDGF | 25 | Control | 2.85 | 2.01 | Inf | 1.41 | 0.157 |
| Fecundity (media treatment; 25 °C) |  |  |  |  |  |  |  |  |
|  | **Contrasts** | **Temperature** | **Media Treatment** | **estimate** | **SE** | **df** | **t.ratio** | **P** |
|  | HD_Control - HDGF | 25 | Control | 2.81 | 1.93 | 11.7 | 1.46 | 0.171 |
|  | HD_Control - HDGF | 25 | Spinosad | -0.456 | 1.93 | 11.6 | -0.236 | 0.817 |

**References**

Rudman SM, Greenblum SI, Rajpurohit S, Betancourt NJ, Hanna J, Tilk S, et al. Direct observation of adaptive tracking on ecological time scales in Drosophila. Science. 2022 Mar 18;375(6586):eabj7484. doi:[10.1126/science.abj7484](https://doi.org/10.1126/science.abj7484)

Gabidulin AR, Rudman SM. MLDAAPP: machine learning data acquisition for assessing population phenotypes. G3 Genes|Genomes|Genetics. 2025 Oct 1;15(10):jkaf173. doi:[10.1093/g3journal/jkaf173](https://doi.org/10.1093/g3journal/jkaf173)

Markow TA, O’Grady P. Reproductive ecology of Drosophila. Functional Ecology. 2008;22(5):747–59. doi:[10.1111/j.1365-2435.2008.01457.x](https://doi.org/10.1111/j.1365-2435.2008.01457.x)

Gibert P, Huey R. Chill‐Coma Temperature in Drosophila: Effects of Developmental Temperature, Latitude, and Phylogeny. Physiological and Biochemical Zoology. 2001 May. Located at: world. doi:[10.1086/320429](https://doi.org/10.1086/320429)

Macdonald SS, Rako L, Batterham P, Hoffmann AA. Dissecting chill coma recovery as a measure of cold resistance: evidence for a biphasic response in *Drosophila* *melanogaster*. Journal of Insect Physiology. 2004 Aug 1;50(8):695–700. doi:[10.1016/j.jinsphys.2004.05.004](https://doi.org/10.1016/j.jinsphys.2004.05.004)

Rako L, Hoffmann AA. Complexity of the cold acclimation response in *Drosophila melanogaster*. Journal of Insect Physiology. 2006 Jan 1;52(1):94–104. doi:[10.1016/j.jinsphys.2005.09.007](https://doi.org/10.1016/j.jinsphys.2005.09.007)

Hallas R, Schiffer M, Hoffmann AA. Clinal variation in Drosophila serrata for stress resistance and body size. Genetics Research. 2002 Apr;79(2):141–8. doi:[10.1017/S0016672301005523](https://doi.org/10.1017/S0016672301005523)

Brooks ME, Kristensen K, Benthem KJ van, Magnusson A, Berg CW, Nielsen A, et al. GlmmTMB Balances Speed and Flexibility Among Packages for Zero-inflated Generalized Linear Mixed Modeling. The R Journal. 2017;9(2):378–400.
